# Betacyanin as Bioindicator Using Time-Temperature Integrator for Smart Packaging of Fresh Goat Milk

**DOI:** 10.1155/2020/4303140

**Published:** 2020-05-01

**Authors:** Souvia Rahimah, Wikeu Malinda, Zaida, Nandi Sukri, Jihan Khairani Salma, Trina Ekawati Tallei, Rinaldi Idroes

**Affiliations:** ^1^Department of Food Industrial Technology, Faculty of Agroindustrial Technology, Universitas Padjadjaran, Bandung, West Java, Indonesia; ^2^Department of Visual Communication Design, Faculty of Creative Industry, Telkom University, Bandung, West Java, Indonesia; ^3^Department of Biology, Faculty of Mathematics and Natural Sciences, Sam Ratulangi University, Manado, Indonesia; ^4^Department of Chemistry, Faculty of Mathematics and Natural Sciences, Syiah Kuala University, Banda Aceh, Indonesia

## Abstract

Smart packaging is a packaging system with embedded sensor or indicator technology, which provides information on the quality of the product, especially perishable foods such as goat milk. One application of smart packaging is to use a time-temperature bioindicator. The purpose of this study was to determine the quality of fresh goat milk during storage at freezing temperatures (−20 ± 2°C) for 31 days and room temperature (25 ± 3°C) for 24 hours using a time-temperature indicator by utilizing a natural dye betacyanin. The method used was descriptive analysis, and the data obtained were processed using the correlation regression test. The samples were observed at freezing temperature every 24 hours and room temperature at 0, 1, 2, 3, 4, 5, 6, 8, 10, and 24 hours. The observation criteria consisted of changes in bioindicator color, milk pH, and total microbes. The results showed that color changes of the bioindicator film at room temperature were more noticeable than at freezing temperature. Based on changes in color of the bioindicator at room temperature, the sample was safe for consumption until the 5th hour with pH 6.51, and the biofilm color characteristics were *L*∗ = 82.49, *a*∗ = 21.46, and *b*∗ = −7.33, but the total number of microbes did not fulfil Indonesian National Standard at the 24th hour, i.e., 1.36 × 10^6^ CFU/ml. At freezing temperatures, fresh goat milk was still safe for consumption until the 31st day with pH 6.51 and total microbe of 1.89 × 10^5^ CFU/ml, and the biofilm color characteristics were *L*∗ = 80.52, *a*∗ = 24.15, and *b*∗ = −7.91. The results of this study concluded that the milk expiration limit based on the betacyanin indicator was 5 hours at room temperature and 31 days at freezing temperature.

## 1. Introduction

Food quality is one of the sensitive issues that always take more attention in the society. Most foods are vulnerable to damage which can be caused by expired preservative, environmental storage, storage time [1, 2], or metal contamination [3, 4]. Several analysis methods or instruments have been introduced to ensure the food quality, including conducting physical assessment [5], the use of chromatography [6], laser [7], electronic sensing [8], biosensor [9, 10], and colourimetric [11]. These methods use an approach of response between instrument and food content such as fatty acid [12, 13], carbohydrate, and protein [14] or degradation products like acidic or basic substances [15, 16].

Although very accurate, the application of these methods has challenges. Chromatographic and colourimetric analysis require laboratory support [17]. *In situ* and real-time methods of laser or sensor are also still ineffective to be applied in food stores or supermarkets that dominantly sell packaged foods [18, 19]. These kinds of foods require a direct indicator on the packaging that provides real-time food quality information. Nowadays, it is well known as smart packaging [20].

Smart packaging is equipped with embedded sensor or indicator technology that can provide information on product's quality [21]. The use of smart packaging is one of the efforts in increasing food security considering that it has the ability to detect damage to products directly and accurately [22]. It can be applied to perishable goods such as fresh goat milk.

In Indonesia, fresh goat milk is generally not given heat treatment after milking because of the low stability of the milk quality against heating [23], so it is immediately frozen. Heat treatment can cause instability of casein, changes in the size of micellar particles, phosphate will be in the aqueous phase [24], faster protein coagulation time, and the decrease of calcium solubility [25].

During storage, milk quality can decrease, showed by a decrease of pH due to microbial activity. In this sense, goat milk quality parameters include pH and the number of microbes which are determined in accordance with Indonesian National Standard (INS). According to INS 2000, the maximum number of fresh milk bacteria is 1 × 10^6^ CFU/ml, and the standard number of bacteria received by the milk processing Industry is less than 5 × 10^6^ CFU/ml. The normal pH value of fresh milk is 6.5–6.7 [26], and the pH standard of fresh goat milk according to Thai Agricultural Standard [27] is 6.5–6.8.

Methods for detecting milk damage due to acid production include the pH indicator [28], measuring the gas concentration in milk using a gas sensor [29], and radio frequency identification (RFID) [30]. These indicators use complex sensor systems and require many electronic components to process signals in making decisions. Because the sensor system requires a large cost, it will affect the selling price of goat milk.

One simple smart packaging indicator is the time-temperature indicator or called the time-temperature integrator (TTI) by utilizing natural dyes as indicators. The dye can change in response to changes of the products during storage. Dyes that can be used are synthetic dyes such as methyl red and bromothymol blue or natural dyes such as red-violet betacyanin and the yellow betaxanthin pigments from red dragon fruit. The peel contains the betacyanin pigment ranging from 115.61 to 118.97 g/kg [31]. Utilization of dragon fruit peel waste as a dye extract can reduce waste production due to increased public consumption of dragon fruit.

Because of the importance of using the right indicators to determine the expiration period of food/beverage products, the purpose of this study was to determine the quality of fresh goat milk during storage at freezing temperatures (−20 ± 2°C) for 31 days and room temperature (25 ± 2°C) for 24 hours using betacyanin from the peel of red dragon fruit as the time-temperature indicator.

## 2. Materials and Methods

### 2.1. Preparation of Fresh Goat Milk

The sample used was freshly milked goat milk. The level of acidity (pH) and total microbes were measured immediately after milking. Then, milk was packaged into 100 ml sterilized bottles. Bioindicator film was affixed to the outside of the bottles, and the bottles were stored at predetermined temperature.

### 2.2. Extraction and Characterization of Dragon Fruit Peel Dye

The extraction and characterization of dragon fruit peel dye followed the procedure provided by Jazira [32] with modification. Dragon fruit peels were sorted and then drained. Approximately 100 g of the sample was crushed using a blender for 5 minutes with distilled water containing 0.2% citric acid, with the ratio of the solvent and the sample which was 1 : 3. The maceration process was performed using a magnetic stirrer for 1 hour. The macerated sample was vacuum filtered, and then the resulting filtrate was centrifuged at 5,000 rpm for 30 minutes. The filtrate was concentrated using a rotary evaporator up to 50% of the initial volume. The resulting pigment extract was observed for total betacyanin, pH, and colour and stored in a dark bottle at a low temperature for further processing.

### 2.3. Preparation of the Bioindicator Film

The procedure for the preparation of the bioindicator film followed Ardiyansyah et al. [33] and Zhai et al. [34] with modification. A total of 1 gram of polyvinyl alcohol (PVA) and 2 grams of tapioca flour were mixed and heated in 70 ml of distilled water using a magnetic stirrer to form homogeneous solution. Then, 30 ml of red dragon fruit peel extract was added and homogenized using a homogenizer at 8,000 rpm for 5 minutes. After that, the bubbles in film solution were removed (degassed) using a sonicator for 5 minutes at room temperature. A total of 30 ml of film solution was poured onto a 9 cm diameter plastic Petri dish and dried using an oven blower at 40°C for 36 hours.

### 2.4. Bioindicator Application in Fresh Goat Milk Packaging

The procedure for application of the bioindicator in fresh goat milk packaging followed Lai et al. [35] and Nofrida [36] with modification. Bioindicator films were placed on the outside of the milk bottles and stored at room temperature (25 ± 2°C) for 24 hours as well as at freezing temperature (−20 ± 2°C) for 31 days. Observations were carried out every 24 hours for the samples stored at freezing temperature and at 0, 1, 2, 3, 4, 5, 6, 8, 10, and 24 hours for the samples stored at room temperature. Observations made included measurements of the bioindicator colour, milk pH, and milk total microbes.

### 2.5. Bioindicator Colour Measurements

Bioindicator colour changes were measured using chromameter CM-5. Samples were fixed on a digital detector that will read the colour of the biofilm. The measurement results were read and completed on the screen (display) stated in CIE *L* ∗ *a* ∗ *b* (with the notation *L*, *a*, and *b*).

### 2.6. pH Value Measurement

Value of milk pH was measured according to Indonesian National Standard (1998). Thawed goat milk was put into a 10 ml measuring cup. The electrode was dipped in milk until pH was stable.

### 2.7. Total Microbe Measurement (Total Plate Count)

Total plate count (TPC) test was carried out according to Indonesian National Standard (1998). The process was done aseptically to prevent undesirable contamination. One millilitre of milk from each sample was homogenized in 9 mL distilled water. One millilitre of each dilution was placed on the Petri dish containing 12−15 ml of molten agar. The media were allowed to solidify for 30 min and incubated at 35°C for 48 hours. The Petri dishes were placed upside down in the incubator. Colonies were counted by using the digital colony counter.

## 3. Results and Discussion

### 3.1. Extraction and Characterization of Betacyanin

The total betacyanin obtained from the extraction of red dragon fruit peel was 119.62 mg/L and pH 3.90. This result was higher than the extract obtained by Jazira [32], which was 81.41 mg/L and pH 3.73. The study of Gengatharan et al. [31] showed that the extraction of red dragon fruit with distilled water produced a total betacyanin from 115.61 to 118.97 g/kg.

Red dragon fruit peel extract prepared in this study had a value of *L*∗ = 30.53, *a*∗ = 65.79, and *b*∗ = 50.21. These results were higher than the results of measurements made by Rachman (*L*∗ = 28.37, *a*∗ = 35.5, and *b*∗ = 7.65) and Widyasanti et al. [37] (*L*∗ = 29.69, *a*∗ = 49.05, and *b*∗ = 32.07). The *L*∗ value indicates the colour brightness of a sample, with a range of values between 0 (dark or black) and 100 (bright or white). If the *L*∗ value is greater or closer to 100, then the sample has a higher brightness level. The value of a∗ is a colour measurement parameter that indicates the degree of redness. *a*∗ + (red) values range from 0 to 100, and *a*∗-(green) values range from 0 to −80. The *b*∗ value is a colour parameter that indicates the degree of yellow. The *b*∗ + (yellow) value is in the range of 0 to 70, and the *b*∗-(blue) value is between 0 and −70.

The total betacyanin pigment in dragon fruit is affected by the betacyanin synthesis process which produces red-purple colour in dragon fruit. The peel of a red dragon fruit changes colour to perfect red with a high total soluble solid content obtained during the best harvesting period [37] because of the pigmentation process during maturation of dragon fruit [39].

### 3.2. The *L*∗ Value of the Bioindicator Film

**Figure 1:**
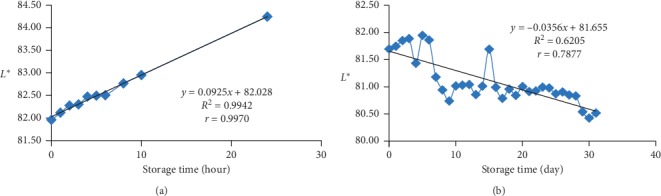
(a) The *L*∗ value of the bioindicator at room temperature (25 ± 2°C). (b) The *L*∗ value of the bioindicator at freezing temperature (−20 ± 2°C).

At room temperature storage, the *L*∗ value in the bioindicator film increased until the 24th hour. This indicates that the colour of the film was fading and approaching white. The bioindicator film stored at freezing temperature decreased gradually until the 31st day, so the colour on the film became darker. The *L*∗ value at room temperature (Figure 1(a)) was influenced 99.70% by storage time with a very strong correlation (*r* = 0.9970). The *L*∗ value at freezing temperature (Figure 1(b)) was influenced 62.05% by storage time with a strong correlation (*r* = 0.7877). Changes in *L*∗ bioindicator values at room temperature occurred more quickly and more visibly than in freezing temperature. The slope value of *L*∗ increased by 0.0925 unit per hour. Meanwhile, the slope value of *L*∗ decreased by 0.0256 unit every 24 hours.

The increase of the *L*∗ value during storage was caused by the process of betacyanin degradation due to the influence of temperature. Betacyanin can undergo hydrolysis of the N=C bond due to exposure to high temperature so that it decomposes to betalamic acid (yellow) and cyclo-DOPA 5-0-glycosides (colourless) [40]. The *L*∗ value which decreased at freezing temperature was caused by pigment regeneration [41] which caused red colour to appear darker.

### 3.3. The *a*∗ and *b*∗ Value of the Bioindicator Film

Based on observation, a decrease in the value of *a*∗ at room temperature occurred constantly, while at freezing temperature, it did not occur constantly despite an increase. At room temperature, the value of *a*∗ decreased which indicating red colour on the bioindicator film decreased during storage. Meanwhile, the *a*∗ value at freezing temperature increased which means red colour on the bioindicator film increased during storage.

**Figure 2:**
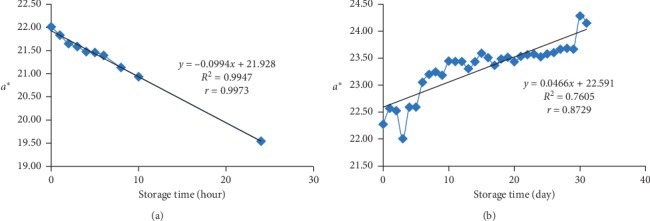
(a) The *a*∗ value of the bioindicator at room temperature (25 ± 2°C). (b) The *a*∗ value of the bioindicator at freezing temperature (−20 ± 2°C).

The *a*∗ value at room temperature (Figure 2(a)) was influenced 99.47% by storage time with a very strong correlation (*r* = 0.9973). The *a*∗ value at freezing temperature (Figure 2(b)) was affected 76.05% by the storage time with a very strong correlation (*r* = 0.8729). Changes in the value of *a*∗ bioindicator at room temperature are greater than the freezing temperature, which was characterized by a greater percentage of influence. The slope value of *a*∗ at the room temperature decreased by 0.0944 unit per hour. Meanwhile, the slope value of *a*∗ at the freezing temperature increased by 0.0466 unit every 24 hours.

A decrease in the *a*∗ value indicates a decrease in the concentration of betacyanin which was affected by temperature. Reduction of redness degree is caused by an increase in the rate of the structural transformation reaction in the red flavylium cation to a colourless chalcone [42]. Betacyanin pigments tend to be more stable at low temperature. At low-temperature storage, the structure of betacyanin does not divide into isobetanin and neobetanin [43]. In addition, at low temperature, the regeneration process of the betacyanin pigment can increase [44]. After a brief warm-up in the preparation process of the indicator film, betalamic acid and cyclo-DOPA derivatives condensed back to form a new betacyanin pigment, so red colour will increase.

**Figure 3:**
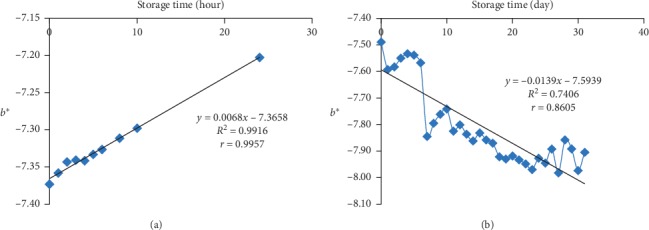
(a) The *b*∗ value of the bioindicator at room temperature (25 ± 2°C). (b) The *b*∗ value of the bioindicator at freezing temperature (−20 ± 2°C).

The *b*∗ value of the bioindicator film at room temperature storage increased which indicates that yellow colour of the bioindicator film was increasing. The *b*∗ value in freezing temperature decreased which means yellow colour on the bioindicator film decreased during storage. The value of *b*∗ at room temperature (Figure 3(a)) was influenced 99.16% by storage time with a very strong correlation (*r* = 0.9957). The *b*∗ value at freezing temperature (Figure 3(b)) was affected 74.06% by the storage time with a very strong correlation (*r* = 0.8605). Changes in *b*∗ value of the bioindicator at room temperature occurred higher than the freezing temperature, which was characterized by a higher percentage of influence. The slope value of *b*∗ at the room increased by 0.0068 unit per hour. Meanwhile, at the freezing temperature, the *b*∗ value decreased by 0.0139 unit every 24 hours.

An increase in the value of the degree of yellow at higher temperature indicates that the colour of betacyanin changed more quickly from red to yellow. Betacyanin was immediately dehydrogenated to neobetanin and decarboxylated to 17-decarboxy-betanin compounds that are orange-red in color [44]. Betacyanin stored at room temperature (25°C) was degraded due to the process of isomeration, decarboxylation, or division, causing red color to gradually decrease and eventually become light brown colour [45].

### 3.4. The °hue Value of the Bioindicator Film

**Figure 4:**
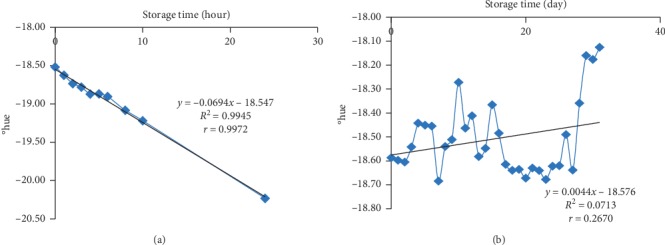
(a) The °hue value of the bioindicator at room temperature (25 ± 2°C). (b) The °hue value of the bioindicator at freezing temperature (−20 ± 2°C).

The °hue is a value that shows the visual degree of colour or the range of colors that can be seen. The value of °hue at room temperature decreased while it increased at freezing temperature. The value of °hue at room temperature (Figure 4(a)) was influenced 99.72% by storage time with a very strong correlation (*r* = 0.9972). The value of °hue at freezing temperature (Figure 4(b)) was only affected 7.13% by storage time with a low correlation (*r* = 0.2670). It indicated that time did not have a major influence on the visual colour change of the bioindicator film.

Changes in the value of °hue seen on the bioindicator film at room temperature were greater than at freezing temperature storage, characterized by a greater percentage of influence and a stronger correlation. The slope value of °hue at the room temperature decreased by 0.0694 unit per hour. Meanwhile, the slope value of °hue at freezing temperature increased by 0.0044 unit every 24 hours. The value of °hue was strongly influenced by the parameter *a*∗ and *b*∗ of the sample. If the value of *b*∗ increases and the value of *a*∗ decreases, the value of °hue will increase, and the chromatic colour ranges from red to yellow [36].

### 3.5. ∆*E* Value of the Bioindicator Film

∆*E* is the total value of the colour change of the sample during storage. The ∆*E* value indicates an overall colour change where a high ∆*E* value indicates that the total colour change of the sample becomes higher during storage. Conversely, a small ∆*E* value indicates that the total change in the colour of the sample during storage is relatively small. The value of ∆*E* in storage at room temperature and freezing temperature increased during storage. This indicates that the bioindicator film decreased in colour intensity so that the final color differed from the original colour.

**Figure 5:**
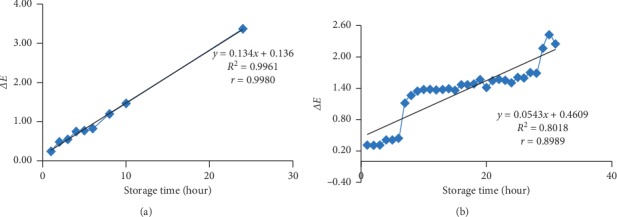
(a) The ∆*E* value of the bioindicator at room temperature (25 ± 2°C). (b) The ∆*E* value of the bioindicator at freezing temperature (−20 ± 2°C).

The value of ∆*E* at room temperature (Figure 5(a)) was influenced 99.61% by storage time with a very strong correlation (*r* = 0.9980). The value of ∆*E* at freezing temperature (Figure 5(b)) was affected 80.81% by storage time with a very strong correlation (*r* = 0.8989). The influence of storage time on changes in ∆*E* at room temperature was higher than at freezing temperature. At freezing temperature, the increase in ∆*E* tended to be lower compared with room temperature. This was caused by low temperature during freezing storage so the colour changes tended to be slower. Slope value of ∆*E* at room temperature increased by 0.134 unit per hour and at freezing temperature increased by 0.0543 unit every 24 hours.

Colour differences can be seen by using a range of numbers based on the total value of the color change (∆*E*). The following are the values used: *E*1 = 0 < ∆*E* < 1: the observer cannot see the difference; *E*2 = 1 < ∆*E* < 2: only trained observers can see the difference; *E*3 = 2 < ∆*E* < 3.5: untrained observers can see the difference; *E*4 = 3.5 < ∆*E* < 5: the difference is very clear and can be seen; and *E*5 = 5 < ∆*E*: the observer is aware of two different colours [46].

During storage at room temperature, the colour change of the bioindicator could not be seen by observers until the 6th hour. In the 8th and 9th hours, only trained observers could see the change in bioindicator film colour. At the 24th hour, untrained observers could see the change in the colour of the bioindicator film. At freezing temperature storage, the colour change of the bioindicator film could not be seen until the 6th day. Meanwhile, from the 7^th^ day to the 28^th^ day, only trained observers could see the change in the colour of the bioindicator film. On the 29^th^ day until the 31^st^ day, untrained observers could see the difference in the colour of the bioindicator film.

### 3.6. Microbiological Quality of Milk

Fresh goat milk is a product that is susceptible to damage due to bacterial activity, considering that no heating process is carried out to reduce microbial growth. During storage, goat milk can undergo physical, chemical, and microbiological changes. Total plate count (TPC) of fresh goat milk on day 0 was 2.26 × 10^5^ CFU/ml, and this is in accordance with Indonesian National Standard SNI No. 01-3141-1998 concerning quality requirements for fresh milk (maximum 1 × 10^6^ CFU/ml) [47] but slightly higher than the standard set by Thailand Agriculture Standard (TAS) No. 6006-2008 (2 × 10^5^ CFU/ml) [27]. At the 8th hour, the total number of microbes approached the maximum limit set by the SNI to 9.76 × 10^5^ CFU/ml, and at the 24th hour, the total number of milk microbes exceeded the SNI set limit which was 1.36 × 10^6^ CFU/ml (Table 1). According to the previous study, TPC of milk can increase to 100 times when stored at room temperature [48].

After being stored for 31 days at freezing temperature, the total microbe dropped from 2.26 × 10^5^ CFU/ml to 1.89 × 10^5^ CFU/ml (Table 2), and the amount was still within the standards set by TAS No. 6006-2008 and SNI No. 01-3141-1998. The decrease in the number of microbes in products stored in extreme freezing temperature is caused by a delay in microbial growth, thereby slowing down the decay of the product [49]. A growth curve describes the process of cell division and the gradual growth of a microorganism from the beginning of growth until the end of activity [50].

**Figure 6:**
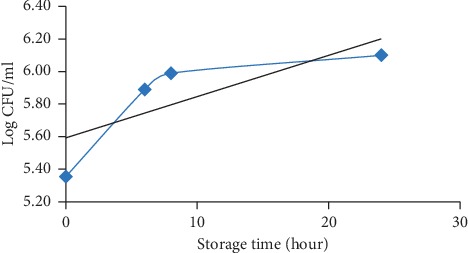
Total microbial growth during room temperature storage (25 ± 2°C).

Figure 6 shows an increase in the number of total microbe population since the beginning of storage so that the adaptation phase could not be determined. At 0 to 6 hours, the total number of microbes increased dramatically, marked by a rapid and constant increase in the curve. Meanwhile, from the 6th hour to the 8th hour, there was only a slight increase in total microbes. The exponential phase is the phase of increasing the activity of deforming and increasing the number of microbes to the maximum speed [51].

From the 8th hour to the 24th hour, the total number of microbes only increased slightly. The stationary phase is a phase of balance where there is an increase in activity and a decrease in microbial activity; in other words, there is a balance between the growing and the mortality rates [51]. In this study, the phase of mortality was undetected because the total microbial count was not continued after 24 hours. The changes in total milk microbes were closely related to the changes in pH values.

**Figure 7:**
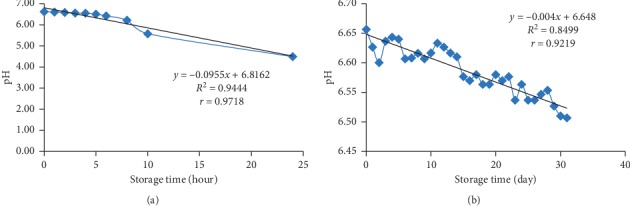
(a) pH of fresh goat milk at room temperature (25°C ± 2). (b) pH of fresh goat milk at freezing temperature (−20°C ± 2).

The pH value of fresh goat milk at room temperature (Figure 7(a)) was affected 94.44% by storage time with a very strong correlation (*r* = 0.9718). The pH value of fresh goat milk at freezing temperature (Figure 7(b)) was influenced 84.99% by storage time with a very strong correlation (*r* = 0.9212). Milk pH value at room temperature and freezing temperature decreased during storage. Milk pH at room temperature at 0 hour was 6.62 and decreased to 6.51 in 5 hours. These results are still in accordance with the standards set by TAS no. 6006-2008 (6.5–6.8). After the 24th hour, pH of milk passed the set limit of 4.49. In freezing temperature, pH of milk was 6.66 on the 0th day to 6.51 on the 31st day, where this decrease still falls within the standard range.

Changes in the pH value of milk at room temperature storage were higher than at freezing storage, indicated by a more significant percentage of influence. The slope value of room temperature storage was −0.0955 so that pH of milk decreased by 0.0955 units per hour. Meanwhile, at the freezing temperature storage, the slope value was −0.004 so that pH of milk decreased by 0.004 units every 24 hours.

The increase in the pH value indicates that the number of lactic acid bacteria was increasing. This indicates that more milk lactose was also converted to lactic acid [52]. Freezing storage condition will inhibit microbial growth [49]. The acid production from the activity of lactic acid bacteria was also inhibited which resulted in a slightly decrease of milk pH.

## 4. Conclusion

At room temperature storage, fresh goat milk was safe for consumption until the 5th hour with a pH of 6.51, colour of the bioindicator film was *L*∗ = 82.49, *a*∗ = 21.46, and *b*∗ = −7.33, and the total number of microbes was 7.73 × 10^5^ CFU/ml. At freezing temperature storage, fresh goat milk was safe for consumption until the 31st day with a pH of 6.51, the total number of microbes was 1.89 × 10^5^ CFU/ml, and colour of the bioindicator film was *L*∗ = 80.52, *a*∗ = 24.15, and *b*∗ = −7.91.

## Figures and Tables

**Table 1:** Total plate count of fresh goat milk during storage at room temperature (25 ± 2°C).

Hour	Total microbe (CFU/ml)	Log CFU/ml
0	2.26 × 10^5^	5.35
6	7.73 × 10^5^	5.89
8	9.76 × 10^5^	5.99
24	1.36 × 10^6^	6.10

**Table 2:** Total plate count of fresh goat milk during storage at freezing temperature (−20 ± 2°C).

Day	Total microbe (CFU/ml)	Log CFU/ml
0	2.26 × 10^5^	5.35
31	1.89 × 10^5^	5.28

## Data Availability

The data used to support the findings of this study are available from the corresponding author upon request.
